# Unveiling the Hemostatic Signature of Prematurity: A Prospective Rotational Thromboelastometry-Based Analysis

**DOI:** 10.3390/medicina61091718

**Published:** 2025-09-21

**Authors:** Martha Theodoraki, Alexia Eleftheria Palioura, Aikaterini-Pothiti Palioura, Abraham Pouliakis, Zoi Iliodromiti, Theodora Boutsikou, Nicoletta Iacovidou, Rozeta Sokou

**Affiliations:** 1 Neonatal Intensive Care Unit, General Hospital of Nikea “Agios Panteleimon”, 18454 Piraeus, Greece; anastasiosmmr@yahoo.gr (M.T.); al.palioura@gmail.com (A.E.P.); kpalioura@gmail.com (A.-P.P.); 2 2nd Department of Pathology, National and Kapodistrian University of Athens Medical School, University General Hospital Attikon, 12462 Haidari, Greece; apou1967@gmail.com; 3 Neonatal Department, National and Kapodistrian University of Athens, Aretaieio Hospital, 11528 Athens, Greece; ziliodromiti@yahoo.gr (Z.I.); theobtsk@gmail.com (T.B.); niciac58@gmail.com (N.I.)

**Keywords:** reference ranges, viscoelastic test, neonatal haemostasis, preterm neonates, haemostatic profile

## Abstract

*Background and Objectives*: The evaluation of the haemostatic mechanism in premature neonates remains particularly challenging, due to their immature haemostatic system, the influence of inflammation and the variety of clinical factors. This prospective study aimed at (a) assessing the haemostatic profile of clinically stable preterm neonates by Rotational Thromboelastometry [ROTEM; (EXTEM, INTEM, FIBTEM assays)], (b) establishing reference ranges, and (c) investigating potential differences in comparison to healthy term neonates. We also evaluated the impact of clinical and perinatal factors on the haemostatic status of this vulnerable population. *Materials and Methods*: 69 premature neonates with no underlying morbidity and 226 healthy term neonates were the study subjects. In term neonates, blood was collected on the 2nd-3rd day of life, if sampling was required for any other reason (hyperbilirubinemia, ABO blood group incompatibility screening, maternal thyroid antibodies, or insufficient prenatal care), whereas in premature neonates, blood was collected between the 4nd-10th day after stabilisation. The parameters measured for each ROTEM assay included Clotting Time (CT), Clot Formation Time (CFT), Alpha angle (α, degrees), Clot Amplitude at 5 and 10 min (A5, A10), Maximal Clot Firmness (MCF), and Lysis Index at 30, 45 and 60 min (Li30, Li45, and Li60 respectively). *Results*: The data analysis demonstrated a prothrombotic profile in preterm neonates, characterized by increased values of A5, A10, (MCF), and α-angle, and shortened CT and CFT across all assays (EXTEM, INTEM, FIBTEM), when compared to term neonates. A statistically significant inverse correlation was observed between gestational age and clot lysis parameters (INTEM Li45, Li60). Additionally, hematocrit levels were negatively correlated with clot amplitude and kinetics of clot development, while platelet count was positively associated with clot firmness parameters (A5, A10, MCF) and α-angle. Mode of delivery and the presence of gestational diabetes did not significantly affect ROTEM assay values. Preterm neonates with a history of respiratory distress syndrome (RDS) exhibited a more pronounced hypercoagulable profile compared to those without RDS, as reflected by the enhanced clot strength and reduced CT, findings that may be attributed to postnatal pulmonary inflammation and its systemic effects on coagulation. *Conclusions*: This study introduces for the first time reference values for the parameters of ROTEM assays (EXTEM, INTEM, FIBTEM) in clinically stable preterm neonates—a highly vulnerable patient group with a distinct need for accurate and individualized monitoring of their haemostatic status. The combined assessment of these assays enhances diagnostic precision, and offers a more comprehensive evaluation of neonatal haemostasis. By defining reference ranges in whole blood, this work provides novel data that support the integration of ROTEM into clinical transfusion algorithms.

## 1. Introduction

Neonatal hemostasis is a dynamic and evolving system that differs fundamentally from that of children and adults [1,2]. The term “developmental hemostasis,” first introduced by Maureen Andrews [3,4] in the 1980s, describes the age-dependent physiological changes affecting both the quantity and quality of hemostatic factors that occur during the maturation process of the hemostatic system from birth to adulthood [5,6,7,8,9,10,11,12]. Although often characterized as “immature,” the hemostatic system of healthy neonates is functionally balanced under normal conditions, without an increased risk of bleeding or thrombosis [4,13,14,15,16,17,18]. However, this balance is fragile, and in cases of severe illness—such as those treated in Neonatal Intensive Care Units (NICUs)—it can be disrupted, leading to significant hemostatic complications [19,20,21,22,23,24,25,26,27,28,29,30,31]. Preterm neonates, particularly those with very low birth weight (VLBW) or who are extremely premature (<28 weeks gestation), have an increased risk of developing hemostatic profile disorders due to the immaturity of their hemostatic system and the higher incidence of aggravating factors, such as hypoxia and sepsis, during their stay in the NICU [28,32,33,34,35,36,37,38]. Additionally, the need for interventions and the inflammatory burden during the early neonatal period further increase the risk of both hemorrhagic and thrombotic complications [29,39,40,41,42].

Bleeding is a common complication in preterm neonates admitted to the NICUs. The risk of severe bleeding, such as intraventricular hemorrhage (IVH), increases significantly with decreasing gestational age (GA) at birth and with the severity of illness during the first days of life [32,33,34,43,44,45,46,47]. In this clinical context, assessment of hemostasis is necessary for the investigation of the cause of bleeding and selection of the appropriate therapeutic approach. Traditionally, the diagnostic approach to coagulation disorders in neonates has included platelet count and conventional coagulation tests, such as prothrombin time (PT), activated partial thromboplastin time (APTT), and fibrinogen levels [48]. There are in vitro plasma-based tests that selectively investigate the involvement of procoagulant factors without assessment of the role of anticoagulant factors or the contribution of cellular elements in hemostasis. These tests generally evaluate isolated parts of the hemostatic mechanism but do not provide information on platelet functionality or the fibrinolytic system [17,32].

However, the hemostatic function in neonates is a dynamically evolving system that involves complex interactions between endothelial cells, plasma proteins, and platelets [49]. During the neonatal period, the concentration of certain procoagulant and anticoagulant factors increases with GA and postnatal age. Consequently, PT and aPTT assays may be inadequate for the evaluation of acquired coagulation disorders, which are common in preterm or critically ill neonates [50,51]. Therefore, the accurate assessment of hemostasis is vital for prevention and individualized management.

Viscoelastic testing (VCT), such as Thromboelastography (TEG), Rotational Thromboelastometry (ROTEM), Sonoclot coagulation and platelet function analyzer, and Viscoelastic Coagulation Monitoring (VCM™), represent a valuable diagnostic approach that is currently best suited to addressing the aforementioned need [45,52]. These methods may also overcome some of the limitations associated with conventional coagulation tests. They provide comprehensive information on the dynamics of clot formation and dissolution, and enable monitoring of all stages of the hemostatic process [53,54]. These tests are performed on a small volume of whole blood and provide data that allow for a comprehensive assessment of the coagulation and fibrinolysis systems. Their results more accurately reflect the in vivo hemostatic function, in accordance with the cellular model of coagulation, taking into account the complex interactions between pro- and anti-coagulant factors, fibrinolytic proteins, cellular components, and platelets—in contrast to the limited information provided by conventional laboratory tests [55].

ROTEM and TEG methods, as point-of-care (POC) techniques for hemostasis assessment, have emerged as valuable tools in the diagnostic approach and personalized hemostatic management of patients with bleeding tendencies [56]. Their clinical application has expanded significantly across various medical fields, including trauma care, cardiac surgery, liver transplantation, and postpartum hemorrhage [57].

Since severe hemorrhages and blood product transfusions are associated with increased morbidity and mortality, the ROTEM-guided hemostatic approach offers distinct advantages [58,59]. It has been documented that ROTEM-guided management reduces transfusion requirements, minimizes complications, lowers hospitalization costs, and contributes to improved clinical outcomes and patient survival [60,61]. Initial assessment using ROTEM is based on the INTEM test (intrinsic pathway, activated with ellagic acid) and the EXTEM test (extrinsic pathway, activated with tissue factor) [62]. Depending on the results, investigation can be further expanded with additional tests: FIBTEM, which isolates fibrinogen function by the inhibition of platelet (PLT) activation (using cytochalasin D); APTEM, which incorporates aprotinin to inhibit hyperfibrinolysis; and HEPTEM, which contains heparinase to detect heparin-induced coagulopathy [62,63].

The use of ROTEM-guided therapeutic protocols can optimize hemostatic strategies, aiming at faster and more accurate interventions, tailored to each patient’s individualized needs. Available data in the international literature regarding the use of TEG and ROTEM methods for the early diagnosis of hemostatic disorders in the neonatal period are limited, especially concerning preterm neonates who are at increased risk for hemorrhagic events [19,20,21,25,26,31,34,64,65,66,67,68,69,70,71,72,73,74,75,76,77,78].

Most studies that attempted to establish reference values for TEG/ROTEM parameters focus primarily on full-term neonates [57,79,80,81,82], while studies involving preterm neonates remain scarce and of limited scope [49,57,65,83]. Furthermore, existing studies often concentrate on individual ROTEM or TEG tests, rather than offering a comprehensive assessment of the neonatal hemostatic profile. This limitation reduces the reliability and clinical applicability of these techniques in transfusion therapy algorithms guided by clinical decision-making. The lack of well-documented reference values—especially for preterm and clinically vulnerable neonates—is a significant gap.

The aim of the present prospective study was to assess the hemostatic profile of preterm neonates at birth by ROTEM, specifically with the EXTEM, INTEM, and FIBTEM tests. The study sought to establish normal reference values for hemostatic parameters in preterm neonates and to explore potential differences in comparison with healthy full-term neonates. Secondary objectives were the evaluation of the influence of sex, mode of delivery, the presence of RDS, and maternal pregnancy-related morbidity on hemostatic balance. The results are expected to provide valuable insight in the understanding of the hemostatic mechanism in preterm neonates and improve the diagnostic accuracy and individualized clinical management of hemorrhagic and thrombotic complications in this particularly vulnerable patient population.

## 2. Materials and Methods

This prospective observational study included healthy stable preterm neonates admitted to the NICU of the General Hospital of Nikaia “Agios Panteleimon,” in Piraeus, between January 2020 and February 2022. The present study is part of a broader research project, the preliminary results of which were published. 226 healthy full-term neonates born at the maternity ward of the same hospital between 2017 and 2020 [79,82] were investigated by ROTEM. The study was conducted in accordance with the principles of the Declaration of Helsinki and was approved by the Scientific and Administrative Board of the General Hospital of Nikaia–Piraeus (Protocol Number: Protocol Number: 3/1; Decision date 25 January 2017). Written informed consent was obtained from the parents of all neonates enrolled in the study.

Healthy preterm neonates were defined as those with a GA less than 37 weeks and an appropriate birth weight for gestational age (AGA), without clinical or laboratory signs of any morbidity. Preterm neonates who, during hospitalization, developed clinical signs consistent with coagulation disorders—such as hemorrhagic tendency, severe bleeding episodes requiring transfusion with blood products [red blood cells (RBCs), plasma (FFP), PLTs], or thrombosis/thromboembolic disease—were excluded from the study. A severe bleeding episode was defined as clinically evident bleeding from any organ system; acute hemorrhage involving any organ (e.g., brain, lungs, stomach, liver); hemoglobin drop of at least 2 g/dL within 24 h; need for transfusion with RBCs, PLTs, FFP, or coagulation factor concentrates; or requirement for surgical intervention to achieve hemostasis.

Healthy full-term neonates were defined as those with a GA greater than 36^+6^ weeks and were AGA, born via VD or elective/planned CS, with an unremarkable perinatal history and no clinical complications from birth until discharge. Full-term neonates born via emergency cesarean section or requiring NICU admission were excluded. Detailed information on the inclusion and exclusion criteria were previously published [79,82].

Neonates were excluded from the study if they had congenital malformations, known or suspected major chromosomal abnormalities, perinatal asphyxia, perinatal blood loss, or if they had received a transfusion with blood products prior to blood sampling for ROTEM analysis. In addition, neonates were excluded if they had personal or family history of bleeding disorders, hemolysis, sepsis, signs of perinatal infection, or evidence of perinatal stress—defined as a non-reassuring fetal condition that did not meet the criteria for perinatal asphyxia [84]. All neonates included in the study received intramuscular vitamin K immediately after birth, in line with standard clinical practice.

Blood in the preterm neonates was withdrawn when they were clinically stable, and in the absence of any other morbidity. In neonates with a GA of 32–36^+6^ weeks, sampling was carried out after stabilization and discontinuation of any form of respiratory support, including supplemental oxygen. In neonates with GA < 32 weeks, sampling was performed when they were stable on nasal continuous positive airway pressure (nCPAP) with a fraction of inspired oxygen (FiO_2_) of 0.21 and a positive end-expiratory pressure (PEEP) of 5 cm H_2_O. All these neonates did not receive any pharmacological treatment at the time of sampling (with the exception of caffeine, if indicated), did not require antibiotic therapy, and were on full enteral feeding. Blood samples from preterm neonates were collected specifically between the 4th and 10th day of life, once they were clinically stable and free of other morbidity.

In full-term neonates, blood was collected between the 2nd and 3rd day of life, during routine blood testing, as mentioned above. 900 μL of residual peripheral blood was used for the measurement of TEM parameters.

Blood was analyzed using the ROTEM^®^ delta analyzer (Tem Innovations GmbH, Munich, Germany). Three ROTEM assays were performed: EXTEM, INTEM, and FIBTEM. Whole blood was collected in 0.109 mol/L (3.2%) sodium citrate tubes at a 9:1 blood-to-anticoagulant ratio (*v*/*v*). Samples were carefully inspected for fibrin clots, and any unsuitable specimen was excluded from analysis.

Various ROTEM parameters were measured, including CT (seconds),CFT (seconds), Clot Amplitude at 5 and 10 min (A5, A10), Alpha angle (α, degrees), MCF (mm),Lysis Index at 30, 45, and 60 min (LI30, LI45, LI60, %).

In parallel with the ROTEM analyses, additional blood tests were performed on the study population, including: Complete blood count (CBC), peripheral blood smear, and bilirubin levels. For the CBC the Sysmex XE-2100 analyzer (Roche, Lincolnshire, IL, USA) was used, and bilirubin levels were measured on the EXL DIMENSION analyzer (Siemens Healthcare Diagnostics, Newark, DE, USA). ABO and Rhesus blood groups were documented for all neonates included in the study.

For all neonates enrolled in the study demographic data (GA, birth weight (BW), and sex), physical examination, medications administered, acid-base balance, blood glucose, vital signs (including body temperature), basic physiological functions, feeding method, postnatal day at the time of sampling, and time to achieve full enteral feeding were recorded. Maternal medical history, medications administered during or prior to pregnancy, and history of the index pregnancy were also recorded. In accordance with the standard neonatal care protocols of the unit, particular attention was paid to any signs of clinical destabilization or disruption of hemostatic balance. As our study population consisted of preterm neonates—who frequently present with RDS [85,86]—the need for surfactant administration as part of respiratory support was also recorded.

### Statistical Analysis

Statistical analysis for determining reference values of ROTEM parameters in the study sample was conducted using SAS 9.4 for Windows (SAS Institute Inc., Cary, NC, USA). For all ROTEM assays (EXTEM, INTEM, FIBTEM), median values and corresponding reference intervals (2.5th and 97.5th percentiles) were calculated following NCCLS guidelines (Jones & Barker, 2008) [87]. Data distribution for all ROTEM parameters was evaluated using Q-Q plots and the Shapiro–Wilk test. Since all variables showed a non-normal distribution, non-parametric statistical methods were employed: the Mann–Whitney U test for comparisons between two groups, the Spearman correlation coefficient (r) for assessing associations, and the Kruskal–Wallis test for comparisons among more than two groups. Two-tailed hypothesis testing was applied, with a *p*-value ≤ 0.05 considered statistically significant. Therefore, *p*-values ≤ 0.05 were used to reject the null hypothesis.

## 3. Results

### 3.1. Demographic and Clinical Data of the Enrolled Neonates

This study included 226 full-term and 69 preterm neonates. The median BW was 3300 g (IQR: 3050–3500) for full-term neonates and 2180 g (IQR: 1820–2300) for preterm neonates (*p* = 3.48 × 10^−35^). The median GA was 39 weeks (IQR: 38–40) in full-term neonates and 34 weeks (IQR: 32–35) in preterm neonates (*p* = 9.07 × 10^−38^). The clinical and demographic characteristics of study population are depicted in Table 1.

### 3.2. ROTEM Findings in Preterm Neonates

All parameters of the ROTEM tests (EXTEM, INTEM, and FIBTEM) are presented as median values, means, standard deviations, minimums, maximums, and reference ranges (2nd, 50th, and 97.5th percentiles) in Table 2, Table 3 and Table 4.

Correlation analyses were performed between ROTEM parameters (INTEM, EXTEM, FIBTEM) and the demographic and hematological characteristics of the preterm neonates in the study sample. The results are presented in Table 5, Table 6 and Table 7, respectively.

Statistical analysis demonstrated a significant negative correlation between GA and the fibrin lysis index. Similarly, BW was found to be significantly negatively correlated with the fibrin lysis index, specifically INTEM LI45 and LI60.

Hct values demonstrated a statistically significant negative correlation with A5, A10, MCF, and clot formation speed across all three assays. Additionally, Hct values had a statistically significant positive correlation with CT in the EXTEM and FIBTEM assays, as well as with CFT in the EXTEM and INTEM assays. PLT count had a statistically significant positive correlation with clot amplitude and kinetics of clot development, as indicated by the parameters A5, A10, MCF, and alpha angle (α) across all ROTEM assays.

Mode of delivery and maternal gestational diabetes did not appear to be associated with ROTEM parameter values. Preterm neonates with a history of RDS, compared to those without, demonstrated a hypercoagulable profile, as evidenced by increased clot firmness (A5, A10, MCF) and enhanced clot formation rate (alpha angle) in the EXTEM and FIBTEM assays, as well as A10 in the INTEM assay. Additionally, shortened CT and CFT in the EXTEM and FIBTEM assays further supported this prothrombotic tendency (Table 8).

Furthermore, analysis of the data regarding a history of IVH grade I revealed no statistically significant differences in ROTEM parameters between neonates with and without IVH-I. In the present study, no differences in ROTEM parameters were observed in relation to sex, with the exception of CT in the INTEM assay, which was significantly higher in male neonates [median: 202.5 s (IQR: 191.5–225)] compared to females [median: 192 s (IQR: 169–207), *p* = 0.023], indicating a delayed initiation of the intrinsic coagulation pathway in male neonates.

### 3.3. Comparison of Demographics, Laboratory and ROTEM Parameters Between Term and Preterm Neonates

The analysis of reference values of ROTEM parameters between premature and full-term neonates revealed a hypercoagulable profile in premature neonates, as reflected by the increased values of A5, A10, MCF and alpha angle, as well as shorter CT and CTF times across all ROTEM assays (EXTEM, INTEM, FIBTEM) (Table 9).

A noteworthy finding was the increased fibrinolytic activity in preterm neonates compared to full-term neonates as reflected by the decreased LI60 value in EXTEM. A statistically significant lower haematocrit value was observed in preterm neonates [39.3% (p25–75: 35.7–43.95)] in comparison to term neonates [47.65% (p25–75: 45–51)] (*p* = 3.99 × 10^−18^). Total bilirubin levels were significantly higher in preterm neonates [10 mg/dL (p25–75: 8.2–12.4)] compared to term neonates [8 mg/dL (p25–75: 6–10)] (*p* = 8.26 × 10^−7^). On the contrary, PLT count showed no statistically significant difference between the two groups, with mean values of 285,000/μL (p25–75: 240,000–325,000) in term neonates and 280,500/μL (p25–75: 232,000–372,000) in preterm neonates (*p* = 0.615).

## 4. Discussion

The present study is the first to investigate and establish reference values for the ROTEM parameters (EXTEM, INTEM, FIBTEM) in preterm neonates. Until recently, no reference values were established for the simultaneous application of these three ROTEM assays in the neonatal population, limiting the potential benefit of this method in guiding transfusion therapy. The scarcity of relevant data has hindered the widespread adoption of ROTEM in neonatal clinical practice—particularly among preterm neonates, a population at high risk. One of the major challenges in NICUs is the management of haemostatic dysregulation. These disorders are often serious and life-threatening, and their diagnosis and management is a challenge for Neonatologists. Neonates have specific haemostatic characteristics, which are influenced by GA, vitamin K levels and the degree of hepatic maturity. There are differences in coagulation factors and fibrinolysis among neonates, children and adults [4,88,89,90,91].

Lower levels of coagulation factors in neonates are functionally counterbalanced by lower levels of natural haemostatic inhibitors and deficiencies in fibrinolysis factors [92]. In neonates, haemorrhagic manifestations may range from mild, such as prolonged bleeding at venipuncture sites, to severe or even life-threatening haemorrhagic events [35,38]. The small blood volume, and the difficulty in compensating hypovolaemia, particularly in very premature neonates, render haemorrhagic manifestations of any degree very important for this vulnerable population [93]. Severe bleeding requiring transfusions with adult blood products is associated with increased morbidity and mortality [35,38,94]. Preterm neonates are at increased risk for IVH [95,96] and neonatologists often transfuse blood products in non-bleeding neonates in their agony to protect them from the occurrence of such a condition [97,98,99,100]. The administration of PLTs and/or FFP can be life-saving in cases of active major bleeding; their use, though, in non-bleeding neonates based only on laboratory findings, not only may fail to reduce the risk of hemorrhage, but (particularly PLTs) may increase neonatal morbidity and mortality [101,102,103,104]. The use of conventional coagulation tests, including PT, APTT, PLT count, and fibrinogen assessment, has significant limitations in predicting bleeding and guiding transfusion therapy; taking in consideration the distinct characteristics of the neonatal haemostatic system, the interest has shifted toward newer methods, such as TEG/ROTEM, for the prediction and management of bleeding or thrombotic tendencies in neonates [20,23,76,105,106]. These methods require a small blood volume, provide initial results within 5–10 min, allow prompt identification of the underlying haemostatic disorder, and guide the selection of the appropriate blood product for transfusion [107,108]. Although viscoelastic methods are well established in adults transfusion protocols with improved outcomes [109,110], their use in children, and especially in neonates is limited, as the absence of established reference values is a significant constraint. The current study provides reference values for the parameters of the ROTEM, EXTEM, INTEM and FIBTEM assays in a significant number of healthy full-term neonates and a smaller sample of preterm neonates.

Our study revealed important differences in the haemostatic profile among preterm and full-term neonates. Preterms exhibited characteristics of a prothrombotic tendency, as reflected by the elevated values of the parameters associated with clot size and stability clot amplitude: A5, A10, MCF, as well as by the clot formation speed (α angle). Shorter CT and CFT were recorded, further enhancing a profile of faster haemostatic response in preterm neonates. Increased fibrinolytic activity in preterms in comparison to full-term neonates was exhibited, by the significantly lower values of the LI60 parameter in the EXTEM assay, indicative of a faster clot lysis. This finding is in line with the immaturity of the fibrinolytic system in preterm neonates, as well as with the lower levels of fibrinolysis inhibitors (such as PAI-1) which were previously reported for this population group [111,112,113]. Research findings suggest that, despite the physiological immaturity of their haemostatic system, preterm neonates demonstrate a functionally competent haemostatic profile [29,30]. This observation is particularly important for the reliable and individualised assessment of bleeding or thrombosis risk in ill preterm neonates, and enhances the value of the ROTEM method as a rapid and dynamic tool for the evaluation of haemostasis in the neonatal intensive care setting.

In a previous study by our group [57], reference values and ranges for ROTEM (EXTEM) parameters were defined based on a cohort of 84 healthy preterm and 198 full-term neonates. From the comparison of the two study groups, no statistically significant differences were observed in most ROTEM parameters, apart from decreased LI60 index in preterm neonates. On the contrary, the present study reported clear differences in the haemostatic profile, suggesting potential variations either in the study design or in the characteristics of the study populations. The primary difference between the two studies is the timing of blood collection. In the present study, blood was collected between the 5th to 10th day of life, to assess and establish reference values in clinically stable preterm neonates. These neonates were not receiving any treatment at all, not even antibiotics. In the study by Sokou et al. [57], blood sampling in preterm neonates was predominantly performed between the 2nd and 5th day of life. Changes in the haemostatic profile of premature neonates were recorded, and this may be attributed mainly to the maturation of the haemostatic system [37,89,114]. During the first days of life PLTs number and function are reduced, and haemostatic proteins are immature. By the 10th day of life there is a gradual improvement of these parameters [115]. At the same time, the role of other factors have been recognised, such as the inflammation response associated with prematurity [116], that can reinforce the prethrombotic state, affecting both PLT activity and the synthesis and function of fibrinogen [117,118,119]. This pattern is also reflected in the findings of the present study, where increased values were observed in parameters related to the speed and strength of clot formation (A5, A10, MCF, α-angle), along with CT and CFT. These findings suggest a functionally sufficient, or even hyperactive, haemostatic potential despite the physiological immaturity of the coagulation system at these gestational ages.

This hypothesis is also supported by Raffaeli et al. [65] who provided reference values for the citrated native TEG test in healthy VLBW neonates at birth, and documented the developmental changes in their haemostatic profile during the first month of life. At birth, VLBW neonates exhibited a prolonged CFT and reduced clot strength compared to full-term neonates, with no differences observed in fibrinolysis parameters. Over time, the haemostatic profile shifted toward a more procoagulant phenotype, with shortening of CT and increase in maximum amplitude (MA). Radicioni et al. [83] studied the haemostatic status of neonates using the TEG method in a small sample of neonates, and reported the presence of a prothrombotic phenotype during the early postnatal period. Significant changes were recorded in the basic TEG parameters during the first 21 days of life, such as reduction in reaction time (R) and clot kinetics time (K), as well as increase in the alpha angle and MA. Similar dynamics were also recorded in the classic haematologic tests, with rapid decrease of PT and APTT during the first week of life. Particularly noteworthy are the findings in neonates with IVH, who exhibited increased thrombin-dependent activity on TEG—reflected by reduced R and K values from birth—indicative of a prothrombotic state. The present study did not identify any association between changes in ROTEM parameters and the presence of IVH. It is noted that all IVH cases in the present study were Grade I, which may affect the statistical strength of such correlations. Although haemostatic disorders may aggravate the extent of bleeding [120], the pathogenesis of IVH is mainly attributed to the anatomic and functional immaturity of the vessels in the germinal matrix, the significant fluctuations in cerebral blood flow, and the impaired autoregulation of cerebral circulation in critically ill neonates [121]. Therefore, the haemostatic profile, as assessed by ROTEM, may not be sufficient as a sole indicator for predicting or identifying the risk of IVH, particularly in its mild forms. In a later retrospective study, Motta et al. [49] evaluated the reference ranges of the TEG method parameters in premature neonates, and highlighted the importance of GA in the haemostatic function, as the levels of many haemostatic proteins are directly dependent on GA at birth. TEG parameter values were compared between early preterm and moderate/late preterm neonates, as well as between bleeding and non-bleeding preterm infants. The results showed similar haemostatic profiles between early and moderate/late preterm neonates, which indicates a sufficient haemostatic function independently of GA. The only statistically significant different parameter was the fibrinolytic function, which was increased in early premature neonates. Furthermore, the PLTs count was significantly associated with the alpha angle and the MA of the clot. In line with the findings of our study, Motta et al. [49] reported no statistically significant differences in TEG parameters between bleeding and non-bleeding preterm neonates (with and without IVH).

An interesting finding in our study is the hypocoagulable profile observed in neonates with higher Hct, as reflected by the prolonged CT and CFT, the reduced clot amplitude (A5, A10, MCF), and the slower clot formation rate, a finding that was reported in previous studies. The impact of higher Hct values on TEG/TEM parameters was associated with a hypocoagulable profile in other studies as well [79,82,122], although red blood cells enhance haemostatic function in vivo [123,124]. The increased relative concentration of red blood cells, and the corresponding dilution of fibrinogen and other coagulation proteins in whole blood, has been associated with this finding [125]. Furthermore, the presence of red blood cells seems to affect the structure and mechanical functions of the clot in a manner proportional to their concentration [123]. Other researchers have also shown that low Hct levels in patients with anemia are associated with the hypercoagulable profile of these patients in viscoelastic testing [126,127].

In our study, the PLTs count in term and preterm neonates, as expected, positively correlated with the size and speed of clot formation, and negatively correlated with CT and clot stabilization time in the ROTEM assays, a finding that reinforces the role of PLTs in neonatal haemostasis. These findings are in line with published data in the literature [79,82,128,129].

Regarding the mode of delivery, no differences were observed in ROTEM parameters between neonates born via VD and those delivered by CS. Similar findings were reported by Schott et al. in samples obtained from the umbilical cord, [130] and by Raffaeli et al., who studied TEG parameters in whole blood samples from 153 VLBW neonates [65]. In contrast, Liu et al. [81] reported prolonged CT in the TEG assay in full-term neonates born by CS and in females, compared to those born vaginally and to males. In the present study, no sex-related differences were recorded in ROTEM parameters, except for prolonged CT values in the INTEM assay in male neonates, suggesting a delayed initiation of the intrinsic coagulation pathway in males. This finding is in agreement with certain studies in adults, where females exhibit a hypercoagulable profile, characterized by faster activation of coagulation and increased clot stability [131,132,133], a fact that is partly attributed to the higher concentration of coagulation factors and fibrinogen in females [134,135]. However, data regarding sex-specific haemostatic differences in neonates remain limited, and further studies are needed for the comprehension of the physiology of these differences during the perinatal period. Oswald et al. [136] reported no sex-related differences, similar to those of Raffaeli et al. [65]. Sulaj et al. [137], also reported that NATEM parameters were affected by sex. Male neonates exhibited a hypocoagulable profile compared to females, with prolonged CT and lower values in the A20, MCF, and MCE parameters, which were statistically significant. Theodoraki et al. [79,82], found lower values of INTEM LI45 and LI60 in males, suggesting enhanced fibrinolysis, however, these parameters were not correlated with sex in the present study. Data on sex-specific haemostatic differences in neonates remain limited, and further studies are required to fully understand the physiology underlying these differences during the perinatal period. In our study, no effect of maternal history of diabetes during pregnancy was observed in the neonate’s ROTEM parameters, and these findings are in line with previous reports [57,79,82].

Concerning RDS in neonates, our study revealed a hypercoagulable profile in those with a positive history of RDS. During the acute phase of RDS in very preterm infants, intravascular and extravascular fibrin deposition as well as activation of the coagulation and fibrinolytic systems have been reported [138,139]. Further progression of the haemostatic abnormalities observed during the acute phase of RDS was also reported in neonates with a history of RDS, at 1 and 6 months after the acute phase [140]. In the study by Katsaras et al. [71] changes in the ROTEM parameters were examined in neonates with RDS, through the evaluation of EXTEM, INTEM, and FIBTEM assays in comparison with healthy neonates. Contrarily to our study, their results featured a more hypocoagulable profile (prolonged CT and CFT, and lower A10 values, suggesting delayed clot formation and reduced clot size at 10 min), and elevated fibrinolytic activity (lower LI60 values) in full-term and preterm neonates with RDS in comparison to healthy neonates. These differences may be related to the study design and the characteristics of the study population, as the study population primarily included moderate to late preterm and full-term neonates with mild to moderate RDS. Furthermore, timing of blood sampling was very early, with a median age of 5 h of life (IQR: 5–6.75 h), a time point at which reduced coagulation and increased fibrinolytic tendency were recorded. In our study, blood sampling was performed at a point where the neonates had been stabilized, after the remission of RDS. We believe that during this phase, the changes associated with subclinical postnatal pulmonary inflammation become more apparent [116], which may promote hypercoagulability [141].

Our study, despite its significant contribution to the investigation of haemostatic parameters in preterm neonates, has certain limitations that affect the ability to generalize its findings. The single-center design, the limited number of participants, and the small number of neonates born before 28 weeks of gestation, restricts the ability to fully understand the haemostatic characteristics of this vulnerable group. Additionally, confounding maternal factors and early postnatal therapies cannot be fully excluded, which may have influenced the haemostatic profile of the neonates. Furthermore, the absence of successive measurements of ROTEM parameters during the neonatal period limits the understanding of the dynamic changes in neonatal haemostatic responses. Long-term follow-up data were not collected, and this prevents assessment of the relationship between neonatal haemostatic profiles and subsequent clinical outcomes. However, the absence of this data does not detract from the importance of the study, as it is the first to establish reference values for ROTEM parameters in the EXTEM, INTEM, and FIBTEM assays in this vulnerable population.

## 5. Conclusions

This study offers an innovative contribution to clinical neonatology, as it introduces for the first time reference values for the parameters of the viscoelastic assays ROTEM (EXTEM, INTEM, FIBTEM) in preterm neonates—a patient group with a particular need for accurate monitoring of hemostatic status. The identification of differences in the hemostatic profile between preterm and full-term neonates reinforces the need for a specialized approach in the clinical management of preterm infants.

It is noteworthy that the clinically stabilized preterm neonates exhibited a prothrombotic phenotype, characterized by an increased speed of clot formation. However, increased fibrinolytic activity was also observed, which may lead to an unstable hemostatic state and increase the risk of uncontrolled bleeding or thrombosis. These findings highlight the dynamic nature and complexity of the hemostatic system in preterm neonates, making prompt and individualized monitoring essential. The study reinforces the necessity of implementing viscoelastic tests such as ROTEM as tools for the accurate and immediate assessment of hemostatic status in neonates, demonstrating their reliability for monitoring hemostasis in dynamic and clinically critical situations. The incorporation of these methods into clinical practice, in combination with the new reference ranges, is expected to improve the prognosis and treatment of neonates at increased risk of bleeding or thrombosis. Our data provides the foundation for further research that will contribute to the development of clinical guidelines and monitoring protocols, while also paving the way for future studies that will further investigate the essential hemostatic parameters in preterm neonates. In this way, they will contribute to the improvement of clinical care and treatment for this particularly vulnerable population group.

## Figures and Tables

**Table 1:** Basic Characteristics of the Study Population.

	Variable	Full-Term Infants	Preterm Infants
Neonatal data	Gestational Age (weeks), Median (Q1–Q3)	39 (38–40)	34 (32–35)
	Birth Weight (g), Mean (Q1–Q3)	3297.25 (3050–3500)	2051.74 (1820–2300)
	Sex (Male), *N* (%)	103 (45.75%)	38 (55.072%)
Neonatal laboratory data	Blood Group	A (85/37.61%)	A (13/318.84%)
		B (28/12.39%)	B (20/28.99%)
		AB (5/2.12%)	AB (10/14.49%)
		O (108/47.79%)	O (26/37.68%)
	Rhesus	+195 (86.28%)	+62 (89.86%)
		−31 (13.72%)	−7 (10.14%)
	Hematocrit(%) Median (Q1–Q3)	47.65 (45–51)	39.3 (35.7–43.95)
	Platelet count (Κ/μL) Median(Q1–Q3)	285 (240–325)	280.5 (232–375)
	Total Bilirubin (mg/dL) Median (Q1–Q3)	8 (6–10)	10 (8.2–12.4)
Neonatal Morbidity Data	History of Thrombophilia	None	None
	History of IVH Grade I	None	18(26.08%)
	Respiratory Distress Syndrome	0 (0%)	43(62%)
Pregnancy and Delivery	Medication	None (195/86.28%)	None (65/94.21%)
		Insulin (1/0.44%)	Insulin(1/1.45%)
		Τ4 (27/11.95%)	Τ4 (7/10.14%)
		Heparin LMW (0/0.00%)	Heparin LMW (1/1.45%)
		Norvasc (0/0.00%)	Norvasc (1/1.45%)
		Salospir (1/0.44%)	None
		Prothuril (1/0.44%)	None
	Maternal pregnancy-associated medical condition *Ν* (%)	Gestational Diabetes (9/4.1%)	Gestational Diabetes (7/10.1%)
		Autoimmune Thyroid Disease (2/0.88%)	None
		Hypothyroidism During Pregnancy (24/10.62%)	7/10,14%)
		Graves (1/0.44%)	None
		No Prenatal Checkups (38/17%)	No Prenatal Checkups (0/0.00%)
	Mode of Delivery C-section, *Ν* (%)	78 (34.52%)	63 (91.30%)
	Multiple pregnancies *Ν* (%)	0 (0.00%)	28 (40.58%)
	Smoking *Ν* (%)	20 (8.85%)	0 (0%)

Abbreviations: intraventricular hemorrhage, IVH; Low-molecular-weight heparin, Heparin LMW.

**Table 2:** Reference ranges of EXTEM ROTEM parameters (*N* = 69 in preterm neonates).

Variable	Mean	SD	Min	Max	Median	2.5 Pctl	97.5 Pctl
EXTEM_A05	46.4	7	31	61	48	32	59
EXTEM_A10	55.2	6.8	40	68	56	42	67
EXTEM_CFT	75.6	22.5	40	143	69.5	44	137
EXTEM_CT	48	6.8	33	67	47	37	61
EXTEM_LI30	99.1	1	95	100	99	97	100
EXTEM_LI45	95.8	1.8	89	99	96	92	99
EXTEM_LI60	92.7	2.6	83	96	93	84	96
EXTEM_MCF	59.7	6.1	45	71	60	48	70
EXTEM_alpha	75.3	4.2	64	82	77	65	81

Abbreviations: CT, clotting time (seconds); CFT, clot formation time (seconds); A5, A10, clot strength at 5 and 10 min (mm); MCF, maximal clot firmness (mm); LI30, LI45, and LI60, lysis index at 30, 45, and 60 min (%); Pctl, percentile; EXTEM: extrinsically activated; SD standard deviation.

**Table 3:** Reference ranges of INTEM ROTEM parameters (*N* = 69 in preterm neonates).

Variable	Mean	SD	Min	Max	Median	2.5 Pctl	97.5 Pctl
INTEM_A05	48.1	7	34	61	50	35	61
INTEM_A10	56.5	6.5	43	69	58	44	69
INTEM_CFT	68.1	21	40	126	61	42	119
INTEM_CT	200.5	35.4	133	344	198	141	288
INTEM_LI30	98.4	1.2	95	100	99	96	100
INTEM_LI45	95	2.1	90	99	95	90	99
INTEM_LI60	91.9	3	81	99	92	87	96
INTEM_MCF	59.7	6	47	72	61	47	70
INTEM_alpha	76.4	3.8	66	82	78	68	81

Abbreviations: CT, clotting time (seconds); CFT, clot formation time (seconds); A5, A10, clot strength at 5 and 10 min (mm); MCF, maximal clot firmness (mm); LI30, LI45, and LI60, lysis index at 30, 45, and 60 min (%); Pctl, percentile; SD standard deviation; INTEM: intrinsically activated.

**Table 4:** Reference ranges of FIBTEM ROTEM parameters (*N* = 69 in preterm neonates).

Variable	Mean	SD	Min	Max	Median	2.5 Pctl	97.5 Pctl
FIBTEM_A05	15.5	4.3	7	30	16	7	23
FIBTEM_A10	16.6	4.5	7	31	17	7	25
FIBTEM_CT	45.3	6.2	25	58	45	33	57
FIBTEM_LI30	99.8	0.8	94	100	100	97	100
FIBTEM_LI45	99.7	1.2	92	100	100	96	100
FIBTEM_LI60	99.7	1	94	100	100	97	100
FIBTEM_MCF	18.3	4.9	8	33	19	9	29
FIBTEM_alpha	74.9	6.1	57	86	76	57	84

Abbreviations: CT, clotting time (seconds); CFT, clot formation time (seconds); A5, A10, clot strength at 5 and 10 min (mm); MCF, maximal clot firmness (mm); LI30, LI45, and LI60, lysis index at 30, 45, and 60 min (%); Pctl, percentile; FIBTEM: fibrinogen polymerization; SD standard deviation.

**Table 5:** Correlation table of INTEM parameters with hematological and demographic characteristics of preterm neonates.

	INTEM_CT	INTEM_A05	INTEM_A10	INTEM_CFT	INTEM_MCF	INTEM_alpha	INTEM_LI30	INTEM_LI45	INTEM_LI60
GA	0.04	−0.14	−0.13	0.10	−0.11	−0.10	−0.27	−0.39	−0.59
	0.74	0.27	0.30	0.44	0.37	0.43	**0.02**	**0.00**	**<0.0001**
BW	0.05	−0.11	−0.11	0.07	−0.08	−0.07	−0.21	−0.31	−0.44
	0.70	0.36	0.37	0.56	0.52	0.58	0.09	**0.01**	**0.00**
Hct	0.07	−0.55	−0.53	0.52	−0.51	−0.50	0.13	0.09	0.05
	0.59	**<0.0001**	**<0.0001**	**<0.0001**	**<0.0001**	**<0.0001**	0.30	0.47	0.69
Hb	0.16	−0.52	−0.50	0.51	−0.48	−0.50	0.12	0.05	0.05
	0.20	**<0.0001**	**<0.0001**	**<0.0001**	**<0.0001**	**<0.0001**	0.36	0.69	0.73
PLT	−0.19	0.56	0.57	−0.54	0.58	0.51	−0.31	−0.28	−0.24
	0.12	**<0.0001**	**<0.0001**	**<0.0001**	**<0.0001**	**<0.0001**	**0.01**	**0.02**	0.07
Total BIL	−0.01	0.17	0.15	−0.17	0.13	0.15	−0.10	−0.10	0.13
	0.92	0.17	0.22	0.17	0.30	0.23	0.42	0.44	0.33

Abbreviations: CT, clotting time (seconds); CFT, clot formation time (seconds); A5, A10, clot strength at 5 and 10 min (mm); MCF, maximal clot firmness (mm); LI30, LI45, and LI60, lysis index at 30, 45, and 60 min (%); INTEM: intrinsically activated; Hb, hemoglobin; PLT, platelet; BW, birth weight; GA, gestational age. Each cell shows the Spearman correlation coefficient and below the *p*-value. *p*-values in bold indicate statistical significance.

**Table 6:** Correlation table of EXTEM parameters with hematological and demographic characteristics of preterm neonates.

	EXTEM_CT	EXTEM_A05	EXTEM_A10	EXTEM_CFT	EXTEM_MCF	EXTEM_alpha	EXTEM_LI30	EXTEM_LI45	EXTEM_LI60
GA	0.21	−0.07	−0.08	0.00	−0.05	−0.08	−0.17	−0.24	−0.42
	0.09	0.57	0.51	0.99	0.67	0.50	0.18	**0.05**	**0.00**
BW	0.12	−0.09	−0.10	0.05	−0.07	−0.09	−0.02	−0.08	−0.23
	0.32	0.46	0.45	0.71	0.60	0.45	0.89	0.52	0.08
Hct	0.53	−0.52	−0.49	0.59	−0.48	−0.65	0.13	0.09	−0.03
	<0.0001	<0.0001	<0.0001	<0.0001	<0.0001	<0.0001	0.30	0.46	0.80
Hb	0.44	−0.49	−0.48	0.51	−0.47	−0.55	0.06	0.06	−0.04
	**0.00**	**<0.0001**	**<0.0001**	**<0.0001**	**<0.0001**	**<0.0001**	0.63	0.66	0.76
PLT	−0.39	0.57	0.52	−0.59	0.52	0.46	−0.27	−0.22	−0.14
	**0.00**	**<0.0001**	**<0.0001**	**<0.0001**	**<0.0001**	**<0.0001**	**0.03**	0.08	0.29
TotalBIL	−0.14	0.19	0.20	−0.16	0.15	0.17	−0.17	−0.04	0.08
	0.29	0.13	0.11	0.19	0.23	0.19	0.18	0.75	0.53

Abbreviations: CT, clotting time (seconds); CFT, clot formation time (seconds); A5, A10, clot strength at 5 and 10 min (mm); MCF, maximal clot firmness (mm); LI30, LI45, and LI60, lysis index at 30, 45, and 60 min (%); EXTEM: extrinsically activated; Hb, hemoglobin; PLT, platelet; BW, birth weight; GA, gestational age. Each cell shows the Spearman correlation coefficient and below the *p*-value. *p*-values in bold indicate statistical significance.

**Table 7:** Correlation table of FIBTEM parameters with hematological and demographic characteristics of preterm neonates.

	FIBTEM_CT	FIBTEM_A05	FIBTEM_A10	FIBTEM_MCF	FIBTEM_alpha	FIBTEM_LI30	FIBTEM_LI45	FIBTEM_LI60
GA	0.22	−0.10	−0.15	−0.15	−0.11	−0.23	−0.12	−0.26
	0.08	0.42	0.22	0.24	0.40	0.07	0.36	0.05
BW	0.12	−0.11	−0.16	−0.15	−0.08	−0.14	−0.08	−0.16
	0.34	0.36	0.21	0.24	0.53	0.25	0.53	0.21
Hct	0.50	−0.38	−0.36	−0.36	−0.48	0.07	0.03	−0.05
	<0.0001	0.00	0.00	0.00	<0.0001	0.60	0.80	0.72
Hb	0.44	−0.35	−0.33	−0.34	−0.46	0.10	0.01	−0.07
	0.00	0.00	0.01	0.01	0.00	0.43	0.93	0.60
PLT	−0.26	0.32	0.30	0.29	0.30	−0.11	−0.14	−0.22
	**0.04**	**0.01**	**0.01**	**0.02**	**0.02**	0.37	0.28	0.09
TotalBIL	−0.10	0.25	0.26	0.24	0.18	0.29	0.05	0.10
	0.45	**0.04**	**0.04**	0.06	0.17	**0.02**	0.70	0.46

Abbreviations: CT, clotting time (seconds); CFT, clot formation time (seconds); A5, A10, clot strength at 5 and 10 min (mm); MCF, maximal clot firmness (mm); LI30, LI45, and LI60, lysis index at 30, 45, and 60 min (%); Pctl, percentile; FIBTEM: fibrinogen polymerization; Hb, hemoglobin; PLT, platelet; BW, birth weight; GA, gestational age. Each cell shows the Spearman correlation coefficient and below the *p*-value. *p*-values in bold indicate statistical significance.

**Table 8:** ROTEM parameters in preterm neonates with and without RDS.

	RDS (*N* = 26)					No RDS (*N* = 43)						
Variable	Mean	SD	Median	p25	p75	Mean	SD	Median	p25	p75	Diff	*p* Value
EXTEM_A05	48.7	6.7	50	44	54	44.98	6.9	46	40	49	4	0.03
EXTEM_A10	57.3	6.3	59	52	62	53.85	6.8	55	49	59	4	0.03
EXTEM_CFT	68.2	18.2	62	56	78	80.20	23.8	73	64	97	−11	0.03
EXTEM_CT	44.6	5.8	44	41	47	50.05	6.5	49	45	55	−5	0.00
EXTEM_LI30	99.1	1.0	99	99	100	99.07	1.1	99	99	100	0	0.92
EXTEM_LI45	95.9	1.6	96	95	97	95.80	2.0	96	94	97	0	0.87
EXTEM_LI60	93.2	1.9	93	92	95	92.34	2.9	93	91	94	0	0.36
EXTEM_MCF	61.5	5.6	63	57	66	58.56	6.2	59	54	63	4	0.06
EXTEM_alpha	77.0	3.6	78	77	79	74.34	4.2	76	72	77	2	0.00
FIBTEM_A05	17.0	4.6	17	15	20	14.59	3.9	15	13	17	2	0.04
FIBTEM_A10	18.4	4.5	18	16	21	15.54	4.3	16	13	18	2	0.01
FIBTEM_CT	43.6	5.6	44	39	47	46.41	6.4	46	43	50	−2	0.04
FIBTEM_LI30	100.0	0.0	100	100	100	99.76	1.0	100	100	100	0	0.17
FIBTEM_LI45	99.9	0.3	100	100	100	99.54	1.5	100	100	100	0	0.52
FIBTEM_LI60	99.9	0.3	100	100	100	99.53	1.2	100	100	100	0	0.18
FIBTEM_MCF	20.0	4.7	19	18	22	17.17	4.8	18	14	20	1	0.03
FIBTEM_alpha	77.0	5.8	78	75	81	73.47	5.9	74.5	70	79	3.5	0.01
INTEM_A05	49.8	6.8	51.5	46	54	47.02	6.9	47	41	52	4.5	0.07
INTEM_A10	58.2	6.0	60	55	63	55.41	6.6	56	50	60	4	0.05
INTEM_CFT	64.9	21.6	58.5	50	71	70.12	20.7	66	54	81	−7.5	0.20
INTEM_CT	203.2	43.6	198	183	222	198.88	29.6	197	182	218	1	0.89
INTEM_LI30	98.5	1.4	99	98	99	98.37	1.2	99	98	99	0	0.50
INTEM_LI45	95.3	2.2	96	94	97	94.83	2.1	95	93	96	1	0.32
INTEM_LI60	92.7	2.8	93	91	94	91.32	3.1	92	89	93	1	0.08
INTEM_MCF	61.1	5.5	62.5	58	65	58.80	6.2	60	54	63	2.5	0.07
INTEM_alpha	76.9	4.0	78.5	75	80	76.10	3.7	77	74	79	1.5	0.24

Abbreviations: CT, clotting time (seconds); CFT, clot formation time (seconds); A5, A10, clot strength at 5 and 10 min (mm); MCF, maximal clot firmness (mm); LI30, LI45, and LI60, lysis index at 30, 45, and 60 min (%); Pctl, percentile; ROTEM: rotational Thromboelastometry; EXTEM: extrinsically activated; FIBTEM: fibrinogen polymerization; INTEM: intrinsically activated. Diff: Difference of median RDS vs. non RDS.

**Table 9:** Comparison of ROTEM parameters between term and preterm neonates.

	Term Neonates (*N* = 226)				Preterm Neonates (*N* = 69)					
Variable	Median	p25	p75	IQR	Median	p25	p75	IQR	Diff *	*p* Value
Gestational age (weeks)	39	38	40	2	34	32	35	3	5	<0.0001
Birth weight (gr)	3300	3050	3500	450	2180	1820	2300	480	1120	<0.0001
Hematocrit	47.65	45	51	6	39.3	35.7	43.95	8.25	8.35	<0.0001
PLT (/μL)	285,000	240,000	325,000	85,000	280,500	232,000	372,000	140,000	4500	0.6156
Total bilirubin (mg/dL)	8	6	10	4	10	8.2	12.4	4.2	−2	<0.0001
EXTEM A05	43	39	46	7	48	41	52	11	−5	<0.0001
EXTEM A10	52	48	56	8	56	50	60	10	−4	0.0003
EXTEM CFT	86	73	102	29	69.5	61	87	26	16.5	<0.0001
EXTEM CT	52	46	59	13	47	44	53	9	5	0.0002
EXTEM LI60	95	93	97	4	93	91	94	3	2	<0.0001
EXTEM MCF	59	55	62	7	60	56	64	8	−1	0.2033
EXTEM alpha	73	70	76	6	77	73	78	5	−4	<0.0001
FIBTEM A05	13	11	16	5	16	13	17	4	−3	0.0014
FIBTEM A10	15	12	17	5	17	14	19	5	−2	0.0039
FIBTEM CT	48	42	55	13	45	41	48	7	3	0.0080
FIBTEM LI60	100	100	100	0	100	100	100	0	0	0.3181
FIBTEM MCF	17	14	20	6	19	15	20	5	−2	0.0215
FIBTEM alpha	74	68	77	9	76	72	79	7	−2	0.0040
INTEM A05	44	41	48	7	50	42	54	12	−6	<0.0001
INTEM A10	54	51	57	6	58	50	62	12	−4	0.0004
INTEM CFT	76	64	90	26	61	52	79	27	15	<0.0001
INTEM CT	191	175.5	212	36.5	198	182	221	39	−7	0.1145
INTEM LI60	94	92	95	3	92	90	94	4	2	0.0001
INTEM MCF	59	56	62	6	61	55	64	9	−2	0.2779
INTEM alpha	75	72	77	5	78	74	79	5	−3	<0.0001

Abbreviations: CT, clotting time (seconds); CFT, clot formation time (seconds); A5, A10, clot strength at 5 and 10 min (mm); MCF, maximal clot firmness (mm); LI30, LI45, and LI60, lysis index at 30, 45, and 60 min (%); Pctl, percentile; ROTEM: rotational Thromboelastometry; EXTEM: extrinsically activated; FIBTEM: fibrinogen polymerization; INTEM: intrinsically activated. *: Diff: Difference of medians term vs. preterm.

## Data Availability

The raw data supporting the conclusions of this article will be made available by the authors on request.
